# Enhanced Resting-State Functional Connectivity of the Nucleus Accumbens in First-Episode, Medication-Naïve Patients With Early Onset Schizophrenia

**DOI:** 10.3389/fnins.2022.844519

**Published:** 2022-03-25

**Authors:** Xiaohua Cao, Qiang Li, Sha Liu, Zexuan Li, Yanfang Wang, Long Cheng, Chengxiang Yang, Yong Xu

**Affiliations:** ^1^Department of Psychiatry, First Hospital/First Clinical Medical College of Shanxi Medical University, Taiyuan, China; ^2^Shanxi Provincial Corps Hospital of Chinese People’s Armed Police Force, Taiyuan, China; ^3^Shanxi Key Laboratory of Artificial Intelligence Assisted Diagnosis and Treatment for Mental Disorder, First Hospital of Shanxi Medical University, Taiyuan, China; ^4^Department of Psychiatry, Shanxi Bethune Hospital, Taiyuan, China; ^5^Department of Mental Health, Shanxi Medical University, Taiyuan, China; ^6^Shanxi Provincial Key Laboratory of Brain Science and Neuropsychiatric Diseases, Taiyuan, China

**Keywords:** early-onset schizophrenia, nucleus accumbens, functional connectivity, resting state, functional magnetic resonance imaging

## Abstract

There is abundant evidence that early onset schizophrenia (EOS) is associated with abnormalities in widespread regions, including the cortical, striatal, and limbic areas. As a main component of the ventral striatum, the nucleus accumbens (NAc) is implicated in the pathology of schizophrenia. However, functional connection patterns of NAc in patients with schizophrenia, especially EOS, are seldom explored. A total of 78 first-episode, medication-naïve patients with EOS and 90 healthy controls were recruited in the present study, and resting-state, seed-based functional connectivity (FC) analyses were performed to investigate temporal correlations between NAc and the rest of the brain in the two groups. Additionally, correlation analyses were done between regions showing group differences in NAc functional integration and clinical features of EOS. Group comparison found enhanced FC of the NAc in the EOS group relative to the HCs with increased FC in the right superior temporal gyrus and left superior parietal gyrus with the left NAc region of interest (ROI) and elevated FC in left middle occipital gyrus with the right NAc ROI. No significant associations were found between FC strength and symptom severity as well as the age of the patients. Our findings reveal abnormally enhanced FC of the NAc with regions located in the temporal, parietal, and occipital areas, which were implicated in auditory/visual processing, sensorimotor integration, and cognitive functions. The results suggest disturbed relationships between regions subserving reward, salience processing, and regions subserving sensory processing as well as cognitive functions, which may deepen our understanding of the role of NAc in the pathology of EOS.

## Introduction

Early onset schizophrenia (EOS) is defined as schizophrenia (SCZ) occurring before 18 years of age, which is associated with higher genetic loading (Remschmidt and Theisen, 2012), a more severe clinical course, and poorer outcomes (Immonen et al., 2017). Furthermore, patients with EOS are less affected by chronic medication and environmental factors, which provides a unique opportunity to explore the pathophysiology of SCZ (Vyas et al., 2010).

The neurodevelopmental model (Kochunov and Hong, 2014; Patel et al., 2021) is helpful to understand the pathophysiology of SCZ. Many studies report structural and functional impairments in widespread areas of the brain in EOS, including frontal, temporal, parietal lobe, striatum, thalamus, and limbic regions as well as the cerebellum (Ordóñez et al., 2015; Driver et al., 2020). Among these regions, much attention is drawn to the striatum, possibly because of its central role in the dopamine hypothesis of SCZ (Simpson et al., 2010; McCutcheon et al., 2019).

As a main component of the ventral striatum, nucleus accumbens (NAc) is well-known owing to its important role in the reward circuitry. It is also assumed to be a prime location for elevated dopamine levels and a fundamental region underlying the neural basis of SCZ. NAc receives afferent inputs from limbic areas, including the ventromedial prefrontal cortex, orbitofrontal cortex (OFC), dorsal anterior cingulate cortex (dACC), medial temporal lobe, hippocampus, and amygdala, and sends projections to the globus pallidus, thalamus, and midbrain areas, such as the ventral tegmental area (VTA) and medial substantia nigra (McCutcheon et al., 2019; Robison et al., 2020). As a key hub of the mesolimbic pathway, NAc is involved in the modulation of dopaminergic and glutamatergic activity (Robison et al., 2020) and is identified as the central therapeutic site of action for antipsychotics (Xu et al., 2015; Pan et al., 2016). Furthermore, dysfunction of NAc is implicated in aberrant reward and salience processing as well as reinforcement learning, which may be associated with delusions and negative symptoms of SCZ (Kapur, 2003; Morrison and Murray, 2009).

Several lines of evidence indicate that NAc impairments are associated with the neural underpinnings of SCZ. For example, postmortem analysis reports increased concentrations of dopamine and elevated protein levels of a vesicular glutamate transporter in NAc in SCZ (Bird et al., 1977; McCollum and Roberts, 2015), whereas animal studies find increased dopamine levels and higher glucose uptake in NAc in model rats of SCZ exposed to maternal immune stimulation (Hadar et al., 2015). Besides this, volumetric reduction and altered functional activation levels of NAc were observed in imaging studies of SCZ (Bois et al., 2015; Richter et al., 2015; Huang et al., 2016; van Erp et al., 2016).

Resting-state functional connectivity (FC) is a useful approach to examine temporal correlations between remote brain regions without task demands (Cordes et al., 2001; Hampson et al., 2002). It is considered to reflect local oscillations of neuronal populations (Riedl et al., 2014) and the functional organization of neural networks, making it a relatively useful option to measure the functional role of NAc in the pathology of SCZ. Up to now, there are only a few studies exploring the resting-state FC of NAc in SCZ. Lin et al. (2018) investigated patterns of discrete frontostriatal circuits in first-episode SCZ patients, and altered FC features were only found in the limbic loop with decreased FC between NAc and dACC observed in SCZ. The other three relevant studies chose different clinical samples of SCZ, including SCZ with and without auditory and/or visual hallucinations (Rolland et al., 2015), treatment-resistant SCZ (White et al., 2016), and smokers with SCZ (Potvin et al., 2019). In these studies, alterations in functional connections of NAc in the patient groups are found in regions located in the limbic system (cingulate cortex, insula, parahippocampal gyri, and VTA) and the default mode network (DMN) as well as the prefrontal cortex (middle frontal gyrus) and temporal lobe (superior temporal gyrus).

It should be noted that these studies all focus on adult-onset SCZ, and EOS subjects were not enrolled. The studies investigating neurodevelopment of subcortical areas report linear decreases of NAc volume with age in healthy adolescents (Mills et al., 2014; Wierenga et al., 2014). There is also evidence that healthy children, adolescents, and adults exhibited diverse FC strength of NAc with subcortical regions and frontal midline structures (Fareri et al., 2015; van Duijvenvoorde et al., 2019). Whether the functional connection features of NAc in EOS are different from those of healthy counterparts remains unclear.

Taken together, EOS is characteristic of higher genetic vulnerability and poorer prognosis relative to adult-onset SCZ and is less influenced by environmental adversities, suggesting its unique role in investigating the underlying neural mechanisms of SCZ. The involvement of NAc in the pathology of SCZ is supported by a growing body of evidence, but functional integration patterns of it with other regions in EOS are seldom explored.

In the present study, resting-state, seed-based FC analysis of NAc were performed in first-episode, medication-naïve patients with EOS and healthy controls (HCs), and correlation analyses were done between regions showing abnormal NAc functional integration and clinical characteristics of EOS. Based on the evidence about NAc anatomical and functional features as well as previous imaging studies of EOS, we hypothesized that altered FC of NAc would be found in the frontal lobe and subcortical limbic areas as well as temporal and parietal areas in patients with EOS.

## Materials and Methods

### Participants

Seventy-eight patients with EOS were recruited from the department of psychiatry, First Hospital of Shanxi Medical University, Shanxi China, during a period from January 2010 to April 2019. The diagnosis of SCZ was made independently by two experienced psychiatrists based on the Structured Clinical Interview for DSM-IV-TR, patient version (SCID-I/P). As an interview tool mainly for adults, SCID-I/P does not include the investigation of neurodevelopmental disorders, such as intellectual disabilities, autism spectrum disorders, attention deficit/hyperactive disorder, and tic disorders. Hence, an additional interview with each participant and their guardians were performed independently by two psychiatrists according to the diagnostic criterion for these disorders in DSM-IV-TR to confirm whether the subjects suffered from these disorders.

The inclusion criteria of the patient group were as follows: (1) aged between 6 and 18 years, (2) right-handed, (3) meeting the diagnostic criteria for SCZ according to DSM IV-TR, (4) in the first episode and having a duration of illness less than 1 year, (5) having no history of antipsychotic treatment prior to scanning, (6) having a total score of Positive and Negative Syndrome Scale (PANSS) ≥ 60, (7) a full-scale Intelligence Quotient of more than 70 according to Wechsler Adult Intelligence Scale (for subjects aged more than 16 years) or Wechsler Intelligence Scale for Children (for subjects aged between 6 and 16 years).

A total of 90 age-, gender-, and education-matched HCs were enrolled from the local community through advertisements. Neither the HCs nor their first-degree relatives had a history of neurological or psychiatric diseases.

For both the patient and control groups, the exclusion criteria were as follows: (1) history of any DSM-IV Axis I disorders except for the diagnosis of SCZ in the patient group, (2) having severe physical illnesses currently, (3) any past or current neurological diseases or family history of hereditary neurological disorders, (4) history of head injury with loss of consciousness, (5) alcohol or substance abuse, and (6) contraindications to MRI scans.

The study was approved by the Ethics Committee of the First Hospital of Shanxi Medical University. Informed written consents were obtained from all the participants and their guardians.

### MRI Data Acquisition

MRI data were acquired using a Siemens Trio 3.0 Tesla scanner (Erlangen, Germany). The participants were instructed to stay awake with their eyes closed, remain awake, not think of anything systematically, and keep the head motionless during scanning. After scanning, all subjects reported that they had not fallen asleep or opened their eyes. Functional images were obtained using an echo-planar imaging (EPI) sequence with the following parameters: 32 axial slices; repetition time (TR) = 2,500 ms; echo time (TE) = 30 ms; matrix = 64 × 64; slice thickness = 3 mm with 1 mm gap; flip angle = 90°; field of view = 240 × 240 mm^2^; voxel size = 3.75 × 3.75 × 4 mm^3^. The resting-state scan lasted for 530 s, and 212 volumes were acquired.

### Functional Data Preprocessing

Resting-state functional MRI data were preprocessed using the DPABI^1^ software toolbox. For each participant, the first 10 functional volumes were discarded to ensure equilibration of the magnetic field and adaption of the subjects to the environment of the scanner. The remaining volumes were corrected for slice acquisition and head motion. Six patients with EOS and 11 HCs were excluded because of head motion exceeding 2.5 mm or 2.5° in any direction. The mean frame-wise displacement (FD), which indexes volume-to-volume alterations in head position, was calculated to further remove the effects of head motion (excluding any volume with a mean FD value exceeding 0.5 mm) (Power et al., 2012; Wang et al., 2019). Then, data of the remaining 72 patients in the EOS group and 79 subjects in the control group entered further preprocessing and statistical analysis. Subsequently, the corrected images were normalized into the standard stereotactic EPI template in Montreal Neurological Institute (MNI) space and resampled to a 3 × 3 × 3 mm^3^ resolution. Then, the normalized images were linearly detrended. Nuisance covariates, including Friston 24 motion parameters (Friston et al., 1996), white matter signal, cerebrospinal fluid signal, and whole-brain global signal, were included in a multiple regression model to regress out the effects of signal drifts and non-neuronal blood oxygenation level-dependent (BOLD) fluctuations (Tomasi and Volkow, 2012). Band-pass temporal filtering (0.01–0.08 Hz) was performed to reduce physiological high-frequency noise. Then, spatial smoothing was conducted with an 8-mm isotropic Gaussian kernel for statistical analyses.

### Definition of Region of Interest and Calculation of Functional Connectivity

The bilateral NAc Region of Interests (ROIs) were defined in accordance with the Anatomical Automatic Labeling (AAL3) template (Rolls et al., 2020). Subsequent procedures were performed in left and right ROIs separately. For each of the ROIs, a seed reference time course was computed individually by averaging the time courses of all voxels within the ROI. Subsequently, Pearson’s correlation analyses were performed between the seed reference time course and time series of the whole brain in a voxel-wise way. The resultant correlation coefficients were transformed into *z*-scores by using Fisher’s *z*-transformation to improve normality.

### Statistical Analysis

Statistical analysis of the demographic and clinical data was carried out using the SPSS for Windows (version23.0; SPSS, Chicago, IL, United States). Mann–Whitney *U* test and χ^2^ tests were conducted according to the characteristics of data.

The analyses of imaging data were performed using SPM8^2^ software. One-sample *t-*tests were conducted on the individual *z*-maps of the two groups separately, producing four *t*-maps corresponding to FC patterns of left and right NAc ROIs in both groups. Then two-sample *t*-tests were done for the two ROIs separately to investigate group differences in FC between EOS patients and HCs within the whole-brain mask with age, gender, and mean FD regressed out as covariates. For both one- and two-sample *t*-tests, the Gaussian random-field (GRF) method was applied for multiple comparison correction. A corrected threshold of *p* < 0.05 was considered the criterion for significance with a voxel level threshold of *p* < 0.005 and cluster size > 50 voxels.

Finally, to explore the relationship between clinical, demographic features and the strength of FC, Pearson’s correlation coefficients were calculated between the mean *z*-values of each cluster showing significant group difference and the total score as well as subscale scores of PANSS and age of the patients. A two-tailed *p* level of 0.05 was used as the criterion of statistical significance.

## Results

### Demographic and Clinical Data Comparisons

The demographic and clinical characteristics of the subjects are displayed in Table 1. The age range was 9–17.9 years for the EOS group and 7.5–17.9 years for the HCs. We did not find significant differences in age (Mann–Whitney *U* test, *p* = 0.4591) and gender (χ^2^ test, *p* = 0.2853) between EOS and HCs. However, a significant difference in mean FD (*p* = 0.0008) was observed between the two groups.

**TABLE 1:** Demographic and clinical characteristics of the participants.

Variable	EOS (*n* = 72)	HCs (*n* = 79)	*p* value
	Mean ± SD	Mean ± SD	
Age (years)	14.7 ± 1.78	14.3 ± 2.17	0.4591^a^
Age subgroup (<13/≥13)	7/65	16/63	0.072^b^
Gender (F/M)	48/24	46/33	0.2853^b^
Mean FD	0.12 ± 0.10	0.15 ± 0.08	0.0008^a^
**PANSS**			
Total score	68.9 ± 18.4		
Positive score	15.8 ± 5.19		
Negative score	16.3 ± 7.53		
General	32.9 ± 8.58		

*EOS, early-onset schizophrenia; HCs, healthy controls; Mean ± SD, mean ± standard deviation; F/M, Female/Male.*

*FD, frame-wise displacement; PANSS, Positive and Negative Syndrome Scale.*

*^a^Mann–Whitney U-test.*

*^b^Chi-square test.*

Considering that childhood-onset SCZ is defined as SCZ occurring before 13 years of age (Ordóñez et al., 2015; Driver et al., 2020), we divided the participants into two subgroups according to the age in both groups. One subgroup included subjects aged 13 years and above, and another subgroup included subjects aged under 13 years. As can be seen from Table 1, the sample sizes of the two subgroups were not balanced for both the EOS group and the HCs. In the EOS group, the numbers of subjects aged under 13 and aged between 13 and 18 years old were 7 and 65, respectively. In the control group, the numbers were 16 and 63, respectively. The difference in age distribution did not reach significance in the two groups (*p* = 0.072). As for the obvious disparity between the sample size of different subgroups possibly influencing the efficacy of statistics, we did not perform further two-way analysis of variance (ANOVA) in the imaging data to explore the possible interaction of age and diagnosis.

### Functional Connectivity Pattern of Nucleus Accumbens in Early Onset Schizophrenia and Healthy Controls

**FIGURE 1:**
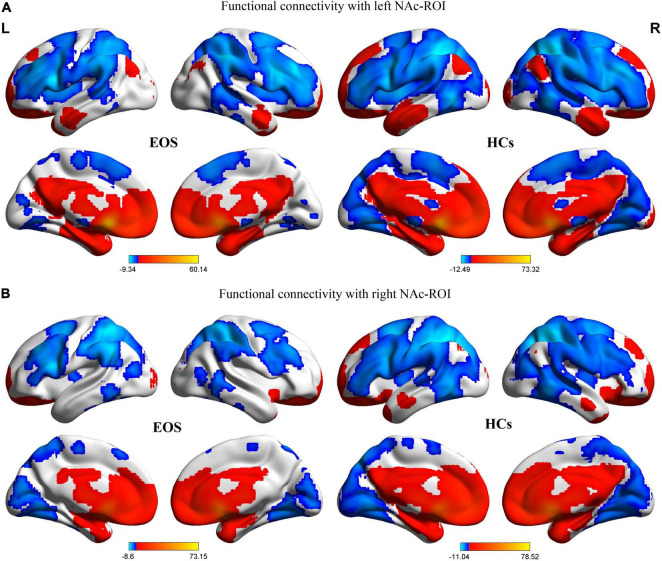
Patterns of significant positive (red) and negative (blue) FC with bilateral NAc ROIs in the patients with EOS and HCs (GRF corrected, voxel level, *p* < 0.005; cluster level, *p* < 0.05; minimum clusters size of 50 voxels). The color bars show the range of *t*-values of the four one-sample *t*-tests, corresponding to FC patterns of the two group. **(A)** FC patterns with the left NAc ROI in the EOS and HCs groups. **(B)** FC patterns with the right NAc ROI in the EOS and HCs groups. FC, functional connectivity; NAc, nucleus accumbens; ROI, region of interest; EOS, early-onset schizophrenia; HCs, healthy controls; L, left; R, right.

The regions showing significant correlations with left and right NAc ROIs in the EOS group and the HCs are shown in Figures 1A,B. In the control group, positive FC with left and right NAc seeds were evident in limbic areas {bilateral NAc, hippocampus, parahippocampa gyrus [Brodmann area (BA) 34], anterior cingulate gyrus (BA 24, 32), insula (BA13), subcallosal gyrus, and amygdala}, medial and inferior parts of the prefrontal cortex [medial/orbital/inferior frontal gyri (BA 9, 10, 11, 47)], striatum (bilateral caudate, putamen and globus pallidus), thalamus, bilateral temporal lobe [superior/middle/inferior temporal gyri (BA 20, 21, 22, 38)], bilateral inferior and medial parietal lobe [inferior parietal lobule (BA 39, 40), posterior cingulate gyrus (BA 23, 31)], and the cerebellum. In the HCs, negative FC with the NAc ROIs was seen in the lateral prefrontal cortex [superior/middle frontal gyri (BA 9, 10, 44, 45, 46)] in both hemispheres, sensorimotor areas [bilateral precentral gyrus (BA 4), postcentral gyrus (BA 1, 2, 3), premotor and supplementary motor area (BA 6), and paracentral lobule], bilateral superior parietal lobule/precuneus (BA 7), occipital lobe [bilateral lingual gyrus, cuneus, superior/middle/inferior occipital gyri (BA 17, 18, 19)], and the cerebellum.

In the EOS group, the spatial distribution of the regions showing significant FC with the NAc ROIs was almost similar to that of the control group. However, as seen in Figure 1, it seems that the range of regions exhibiting significant FC with the NAc ROIs is smaller in the patient group relative to the HCs.

### Group Differences in Functional Connectivity of Nucleus Accumbens

**FIGURE 2:**
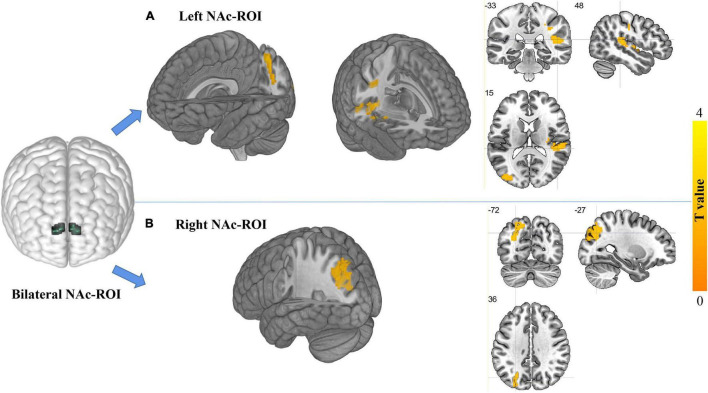
Regions showing differences in FC with the NAc ROIs between groups (GRF corrected, voxel level, *p* < 0.005; cluster level, *p* < 0.05; minimum cluster size of 50 voxels). The locations of bilateral NAc ROIs in the brain are shown in the left part of the figure. **(A)** Regions showing increased FC with the left NAc ROI in the EOS group relative to the HCs. The cluster located in the crosshair corresponds to the right superior temporal gyrus, and another cluster without the crosshair corresponds to the left superior parietal gyrus. **(B)** Regions showing increased FC with the right NAc ROI in EOS relative to the HCs. The cluster located in the crosshair corresponds to the left middle occipital gyrus.

For the left NAc ROI, the EOS patients exhibited significantly increased FC in the right superior temporal gyrus (STG) and left superior parietal gyrus (SPG) when compared with the HCs (*GRF* corrected; voxel level *p* < 0.005, cluster level *p* < 0.05, minimum clusters size of 50 voxels, Figure 2A and Table 2). For the right NAc ROI, significantly increased FC was seen in the left middle occipital gyrus (MOG) in patients with EOS relative to the control group (*GRF* corrected; voxel level *p* < *0.005*, cluster level *p* < 0.05, minimum clusters size of 50 voxels, Figure 2B and Table 2).

**TABLE 2:** Group differences in functional connectivity of bilateral NAc-ROIs between EOS and HCs (EOS>HCs).

Brain regions	Side	BA	Cluster size (voxels)	MNI coordinates (mm)			*t*-value (peak)
				x	y	z	
**Left NAc-ROI**							
Superior temporal gyrus	R	41	322	51	−30	12	3.92
Superior parietal gyrus	L	7	475	−18	−69	48	3.77
**Right NAc-ROI**							
Middle occipital gyrus	L	19	221	−30	−72	30	3.33

*Threshold was set at a voxel level p < 0.005, minimum clusters size of 50 voxels and a cluster level p < 0.05.*

*NAc, nucleus accumbens; ROI, region of interest; BA, Brodmann area; MNI, Montreal Neurological Institute; L, left; R, right.*

### Correlation Analysis

No significant associations were found between the strength of FC in regions showing group differences and PANSS scores and age of the patients with EOS (*p* > 0.05, uncreated Supplementary Figures 1, 2).

## Discussion

In the present study, resting-state FC patterns of NAc were explored in first-episode patients with EOS as well as healthy children and adolescents. The regions exhibiting functional connections with the NAc were observed in a relatively large sample of healthy children and adolescents. For the control group, positive FC with the NAc ROIs were seen in widespread areas, including bilateral limbic regions, striatum, thalamus, medial/orbital/inferior prefrontal cortex, lateral temporal lobe, inferior/medial parietal cortex, and the cerebellum. Meanwhile, negative FC with the NAc ROIs were evident in the lateral prefrontal cortex, sensorimotor areas, superior parietal cortex, occipital lobe, and cerebellum in the HCs. Furthermore, group comparison revealed significant increased FC in the right STG and left SPG with left NAc ROI as well as increased FC in the left MOG with right NAc ROI in patients with EOS. These findings might provide new insight into the neural mechanism of EOS from the perspective of NAc FC.

To our knowledge, this is the first study focusing on FC patterns of NAc in early onset patients with SCZ. Quite a few studies utilize reward-related tasks to explore the functional state of NAc in SCZ. In these studies, both increased (Richter et al., 2015; Li et al., 2018) and decreased (Radua et al., 2015; Huang et al., 2016) activation levels of the patient group were reported. The inconsistency may be due to varieties in cognitive, emotional, and motivational aspects of the paradigms. On the contrary, resting-state FC analysis is helpful to clarify brain activities without taking task demands into account. Besides this, because EOS patients, especially those with a short duration of illness, are less affected by environmental adverse factors when compared with adult-onset counterparts, EOS is possibly more suitable to reflect the neurobiological basis of schizophrenia. Thus, to better deepen our understanding of the functional features of NAc in SCZ, we chose a sample of first-episode, medication-free EOS patients and age- and gender-matched controls and used the approach of FC analysis.

Findings of positive FC with the NAc ROIs were consistent with previous studies about anatomical connections of NAc. According to the evidence from primate research and human studies (Postuma and Dagher, 2006; Cauda et al., 2011; Haber, 2016), NAc receives cortical afferent projections from OFC, medial prefrontal cortex (MPFC), and ACC and has anatomical interconnections with subcortical areas, including hippocampus, amygdala, thalamic nuclei, ventral pallidum, VTA, and substantia nigra as well as other components of the corticostriatal loops, such as caudate and putamen. These areas were largely overlapped with our results. Additionally, our study finds that positive FC with the NAc ROIs were also seen in the insula, parahippocampal gyrus, subcallosal gyrus, inferior frontal gyrus, superior/middle/inferior temporal gyri, posterior cingulate, and inferior parietal lobule, which were in accordance with the studies of NAc FC patterns (Di Martino et al., 2008; Cauda et al., 2011) in healthy volunteers. In the present study, most of the regions showing positive functional correlations with the NAc ROIs (including OFC, MPFC, ACC, hippocampus, amygdala, thalamus, globus pallidus, insula, parahippocampa gyrus, and subcallosal gyrus) are components of the reward circuitry or belong to the limbic striatal loop of the cortical–striatal–thalamic parallel pathways (McCutcheon et al., 2019). These results confirm the central role of NAc in reward-related processing in healthy children and adolescents. Interestingly, some of the regions in our results, including the MPFC, posterior cingulate, inferior parietal lobule, lateral temporal cortex, hippocampus, and parahippocamal gyrus, were all components of DMN. There is evidence indicating that functional and structural alterations in DMN may contribute to the severity of anhedonia in SCZ (Lee et al., 2015; Brakowski et al., 2020). The role of DMN in self-referential processing is well-established (Buckner et al., 2008), and abnormalities of DMN may lead to disruption in self-reflection and self-consciousness, which may result in anhedonia in SCZ. Synchronous, low-frequency spontaneous brain activity during the resting state between NAc and the DMN may confirm the common role of these regions in the processing of pleasure experience, and impairments of them may underlie negative symptoms, including anhedonia in patients with SCZ.

In our study, negative FC with the NAc ROIs were evident in the lateral prefrontal cortex, sensorimotor areas, superior parietal lobe, occipital cortex, and cerebellum. These findings were consistent with a previous study exploring FC patterns of striatum subregions (Di Martino et al., 2008), in which these regions found in our study were all reported to show negative FC with NAc. Although the nature of negative correlations between brain areas or networks remains unclear, several lines of evidence confirm the validity and functional significance of anticorrelations between brain networks (Parente et al., 2018; Parente and Colosimo, 2020). The phenomenon of anticorrelations in the resting-state brain is frequently observed between DMN and task-positive regions (Fox et al., 2005; Uddin et al., 2009) and is considered to reflect opposite goals or functions in brain networks. In the current study, the resting-state spontaneous fluctuations in the NAc, which is a key region in affective modulation and reward processing, were in an antiphase relationship with fluctuations in the regions involved in motor/sensory processing and cognitive control (including the dorsolateral prefrontal cortex, sensorimotor areas, and parietal and occipital cortices). This result is in accordance with the point that anticorrelations exist between networks implicated in different functions.

In the present study, increased FC was found between the left NAc ROI and right STG in the EOS group. The STG contains the primary auditory cortex and Wernicke’s area, which is linked to auditory processing (Mesgarani et al., 2014) as well as production, interpretation, and self-monitoring of language (Ferstl et al., 2008). Volumetric reduction and cortical thinning of STG have been found in SCZ, which is associated with various symptom dimensions, including hallucinations, thought disturbances, and delusions (Walton et al., 2017; Sumner et al., 2018; Bandeira et al., 2021). Additionally, functional abnormalities of STG in SCZ are also confirmed by recent meta-analysis and studies (Li et al., 2019; Zhang et al., 2019). Notably, morphological and functional alterations of STG were also observed in children and adolescents suffering from SCZ (Matsumoto et al., 2001; Li et al., 2015; Liu et al., 2018). Although direct anatomical connection between NAc and STG is not found, a previous study reports increased FC between NAc and STG in a group of SCZ patients with auditory hallucinations when compared with the patients without hallucinations (Rolland et al., 2015). A recent systematic review found structural alterations of STG and NAc are both associated with thought disorder, which is considered a hallmark of SCZ symptomatology (Sumner et al., 2018). This evidence suggests that STG and NAc may be involved in special symptom dimensions of SCZ simultaneously, such as hallucination and thought disorders. Further research work exploring the relationship of STG and NAc in clinical subgroups with various symptom characteristics of EOS is expected.

For the left NAc ROI, the patient group also showed increased FC with left SPG. The parietal cortex, especially the SPG, is implicated in multiple sensory and cognitive functions, including sensorimotor integration, spatial perception, visuospatial attention, and memory (Molholm et al., 2006; Wang et al., 2015). Reduced volume of SPG has been found in adult-onset SCZ (Zhou et al., 2007; Kong et al., 2015; Abrol et al., 2017) and EOS (Thompson et al., 2001; Kumra et al., 2012). Notably, dysregulated activation of SPG was observed in SCZ patients when performing attention and memory tasks (Koch et al., 2010; Li et al., 2012; Kim et al., 2015). Furthermore, abnormal structural and functional changes of SPG were associated with symptom severity (Henseler et al., 2010; Zhao et al., 2018), attention impairment (Kumra et al., 2012), and neurological soft signs (Kong et al., 2015) in SCZ. There were several findings indicating a functional relationship between SPG and NAc. For example, the SPG and NAc were both activated when healthy adolescents were performing risk-taking tasks (Vorobyev et al., 2015), and increased FC between SPG and NAc is associated with displeasure ratings of young adults when watching violent videos (Porges and Decety, 2013). Additionally, increased FC from baseline to a 6-week “reward exposure”–based therapy between NAc and SPG was associated with improvement of depressive symptoms in patients with late-life depression (Solomonov et al., 2020). The NAc is a key component in reward processing, whereas SPG is involved in cognitive functions, such as visuospatial attention, increased FC between NAc and SPG may reflect an alteration pattern of the balance between the reward circuitry and the goal-directed attention networks in SCZ.

The patient group also showed increased FC between right NAc ROI and left MOG. The occipital cortex is well-known for its role in visual processing, and its abnormalities are found to be associated with visual hallucination, object-recognition deficits, and visual perception disturbance (Onitsuka et al., 2007; Silverstein and Keane, 2011) in SCZ patients. Particularly, the MOG belongs to the visual dorsal stream, which is implicated in early visual detection and visual-spatial processing (Wandell et al., 2007). Except for volumetric alterations of MOG (Cooke et al., 2008; Faget-Agius et al., 2015; Singh et al., 2015), functional abnormalities of MOG are reported in adult patients with SCZ (Yu et al., 2013; Singh et al., 2015; Gong et al., 2020) and EOS (Kyriakopoulos et al., 2012; Jiang et al., 2015). Besides this, we note that the activation of MOG was related to the visual perception of emotions, and a higher activation level of occipital areas, including MOG, may indicate increased attention to salient information (Norris et al., 2004). In other words, emotional clues may provide more salient information and enhance the neural activities of MOG. In accordance with this point, altered activation of MOG was found during emotional processing and social perception with affective contents as well as empathy tasks in SCZ (Bjorkquist and Herbener, 2013; Singh et al., 2015; Larabi et al., 2018). Additionally, the activation levels of MOG during object perception were linked to affective symptoms of SCZ (Stephan-Otto et al., 2016). These findings suggest a possible role of MOG in emotional regulation deficits in SCZ, which may be associated with negative and affective symptoms. In the meantime, coactivation of NAc and MOG were found in healthy participants when performing risk-taking decision making (Matthews et al., 2004) and visual vigilance tasks (Breckel et al., 2011). Given the vital role of NAc in salience processing and its anatomical and functional connection with the emotion-regulation circuitry, we postulate that abnormal FC between NAc and MOG may indicate impairments in the visual processing of emotional contents and salient information in SCZ.

In our study, only enhanced FC were found in the EOS group relative to the controls. Hyperconnectivity is considered to reflect increased functional integration of certain regions or compensatory changes in response to a primary deficit (Fornito et al., 2012). In the present study, the regions showing abnormally elevated functional connectivity with the NAc ROIs were located in the temporal, parietal and occipital cortices. As mentioned, these regions are implicated in diverse functional domains, including reward and salient processing, auditory and visual processing, and sensorimotor integration as well as cognitive functions, such as attention and memory. Enhanced FC of NAc with the regions may indicate a compensatory mechanism for the disrupted function of these domains.

The regions showing group differences in our findings did not overlap with the results of previous studies of NAc FC analysis in SCZ (Rolland et al., 2015; White et al., 2016; Lin et al., 2018; Potvin et al., 2019). Previous studies report abnormal FC of NAc with regions located in the limbic system, DMN, and frontal and temporal lobes, whereas our study reveals abnormal FC of NAc in the temporal, parietal, and occipital cortices. The discrepancy may be mainly due to differences in the clinical features of the subjects. The patient groups of the previous studies were first-episode SCZ, SCZ with and without hallucinations, smokers with SCZ, and treatment-resistant SCZ, respectively, all of which were adults. It is likely that EOS exhibits distinct NAc functional integration patterns as compared with adult patients. Future studies with a more refined definition of the clinical characteristics of EOS will be helpful to clarify the functional role of NAc connectivity and the corresponding clinical implications.

In the present study, we did not find associations between FC strength of the regions showing group differences and the PANSS scores as well as the age of the patients, suggesting that altered FC patterns of NAc in the EOS group were independent of the severity of clinical symptoms. In previous studies examining NAc FC in SCZ, altered FC strength of NAc with VTA, dACC, precuneus, and middle frontal gyrus in the patient groups were correlated with the complexity of hallucination (Rolland et al., 2015) and symptom severity (White et al., 2016; Lin et al., 2018), respectively. A possibility accounting for the inconsistency between the above studies and our results is that FC between NAc and distinct regions may take on different roles in the onset and development of SCZ, corresponding to diverse patterns of association with clinical phenotypes. Furthermore, we speculate that functional connectivity characteristics of NAc may be a trait indicator of EOS, which were not influenced by clinical variables of the patients. Some evidence indicates that the functional state of NAc may be a vulnerable mark of SCZ. For example, reduced fractional anisotropy (FA) in the tract connecting the left NAc and left dorsolateral prefrontal cortex (DLPFC) (de Leeuw et al., 2015) as well as decreased FC of the right NAc with right OFC and left middle cingulate cortex (Peeters et al., 2015) are found in both SCZ patients and unaffected siblings of the patient groups. It should be noted that the participants in these studies were all in adulthood, and the regions showing altered structural or FC patterns did not overlap with the results of the present study. Even so, these findings suggest the possible role of NAc function as a trait marker of SCZ. Given that the evidence about this point is not abundant, the interpretation of the null results of correlation analysis should be made with caution. Future research work recruiting participants, such as individuals at ultra-high risk (UHR) for psychosis and relatives of SCZ, may be helpful to clarify this issue.

In our study, the age range of the subjects covered both childhood and the adolescent period. Children and adolescents are in the process of brain maturation, and whether the two age groups exhibited distinct FC patterns remains unclear. Research on healthy volunteers reports age-related decreases in FC between NAc and medial prefrontal, subcallosal regions as well as age-related increases in FC between NAc and ventral ACC (Fareri et al., 2015; van Duijvenvoorde et al., 2019), suggesting dynamic changes in NAc functional integrations. However, we did not find studies that directly compared functional imaging measurements between childhood- and adolescent-onset SCZ. For the patients recruited in our study, the proportion of childhood-onset SCZ was much lower than that of the adolescent-onset counterparts, which is consistent with the fact that childhood-onset SCZ is quite rare relative to adolescent-onset SCZ. Because of the discrepancy in sample size in different age subgroups, we did not perform ANOVA to examine the interaction between age and diagnosis. Hence, the adolescent participants may contribute more to the results of the present study. Further research with comparable sample sizes in both childhood and adolescent age groups is merited to clarify the effect of brain maturation on EOS.

Several limitations of the present study should be considered. First, the current study was performed using a cross-sectional design. Thus, we cannot infer whether the altered FC patterns of the NAc ROIs occurred prior to or after the onset of SCZ and whether it changes dynamically during the progress of the illness. Further longitudinal studies enrolling UHR individuals and EOS patients are needed to explore the dynamic changes of NAc FC. Second, the possible diagnosis of comorbid neurodevelopmental disorders was determined according to DSM-IV, and a diagnostic interview specific for subjects aged under 18 years, such as the Kiddie-Schedule for Affective Disorder and Schizophrenia (K-SADS), were not used. Thus, the process of the exclusion of comorbid neurodevelopmental disorders, which were not included in SCID, may not be rigorous enough. Third, we did not collect data on illness duration and other relevant information, such as cognitive functions and psychosocial factors, which limit the ability to examine the relationships between neuroimaging variables and data of other dimensions. Additionally, the ROI of NAc were generated as a whole and were not separated into subregions of the core and shell. Future studies with a definition of the NAc-ROI subregions were expected to describe FC patterns of NAc in a more refined way. Finally, in the preprocessing steps, functional images of the participants were coregistered to the MNI adult brain template, not to a template generated from children and adolescent data, which may impact the effect of spatial normalization and subsequent analysis to some extent. Although previous studies show that the inconsistency between using an adult and a children’s template during preprocessing would not affect the results of the fMRI analysis significantly (Burgund et al., 2002; Kang et al., 2003; Alexander-Bloch et al., 2010), application of a participant-specific brain template when performing the coregistration step is merited.

In summary, our study reveals enhanced FC strength of the NAc in EOS patients with the regions exhibiting group differences relative to the controls located in the temporal, parietal, and occipital areas, which were implicated in auditory and visual processing and sensorimotor integration as well as cognitive functions. The elevated functional connections of NAc with these regions indicate dysregulated relationships between regions subserving reward and salience processing and regions subserving sensory processing as well as cognitive functions, which may shed light on the understanding of the neural basis of EOS from a perspective of brain FC.

## Data Availability Statement

The raw data supporting the conclusions of this article will be made available by the authors, without undue reservation.

## Ethics Statement

The studies involving human participants were reviewed and approved by the Ethics Committee of the First Hospital of Shanxi Medical University. Written informed consent to participate in this study was provided by the participants’ legal guardian/next of kin.

## Author Contributions

YX designed and supervised the study. XC and QL organized the experimental data. QL performed the statistical analysis. XC wrote the first draft of the manuscript. QL and SL contributed to the revision of the manuscript. ZL, YW, LC, and CY assisted in recruiting the participants. All authors discussed the results and commented on the manuscript.

## Conflict of Interest

The authors declare that the research was conducted in the absence of any commercial or financial relationships that could be construed as a potential conflict of interest.

## Publisher’s Note

All claims expressed in this article are solely those of the authors and do not necessarily represent those of their affiliated organizations, or those of the publisher, the editors and the reviewers. Any product that may be evaluated in this article, or claim that may be made by its manufacturer, is not guaranteed or endorsed by the publisher.

## Footnotes

1
http://rfmri.org/dpabi

2
www.fil.ion.ucl.ac.uk/spm/software/spm8
