# Supplementary figures and images for: Enhanced Resting-State Functional Connectivity of the Nucleus Accumbens in First-Episode, Medication-Naïve Patients With Early Onset Schizophrenia
Source: Front Neurosci. 2022 Mar 25;16:844519. doi: 10.3389/fnins.2022.844519 (PMC8990232; doi:10.3389/fnins.2022.844519)

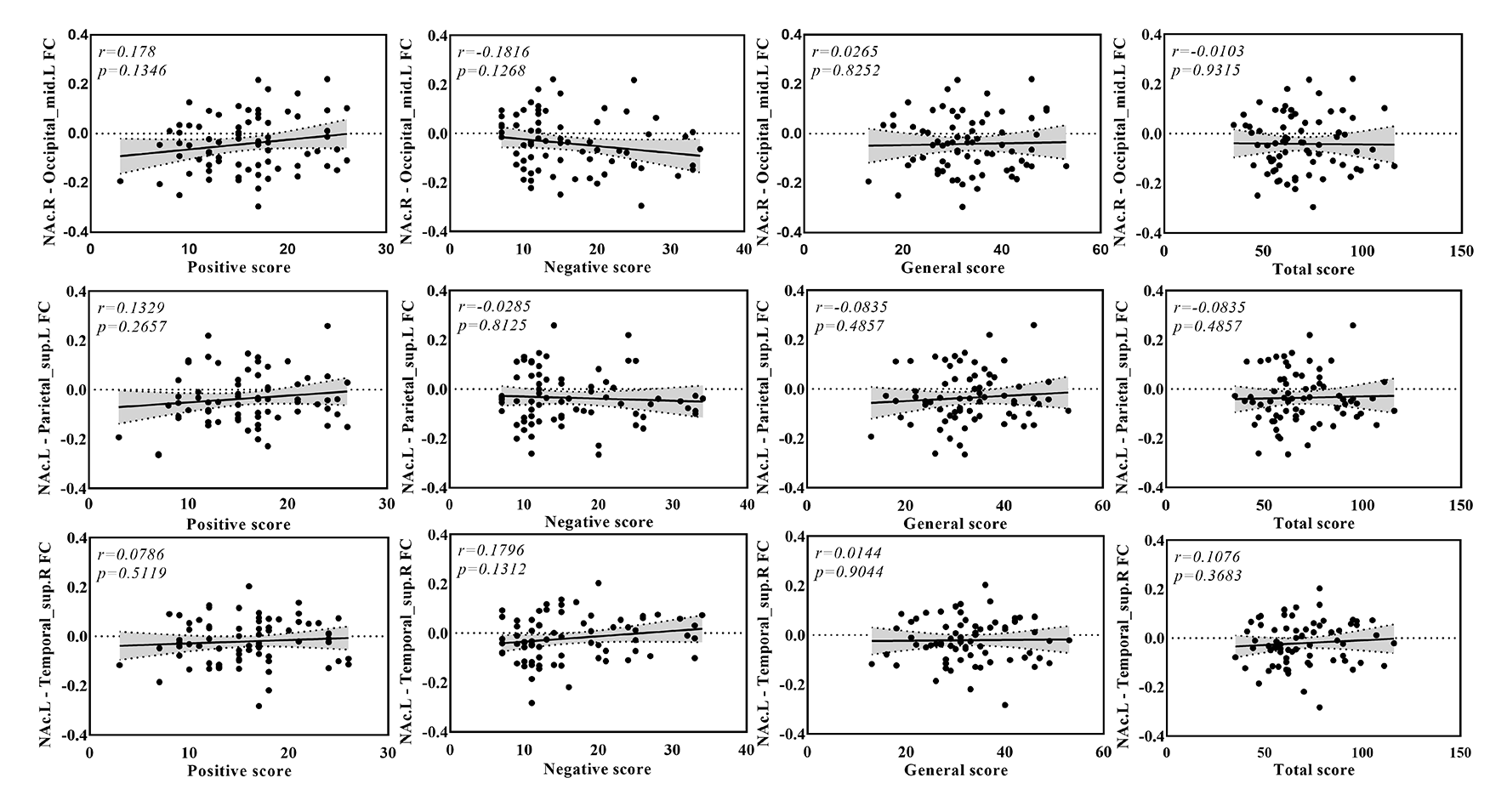

Supplement: Supplementary file 1 [file Image_1.TIF]

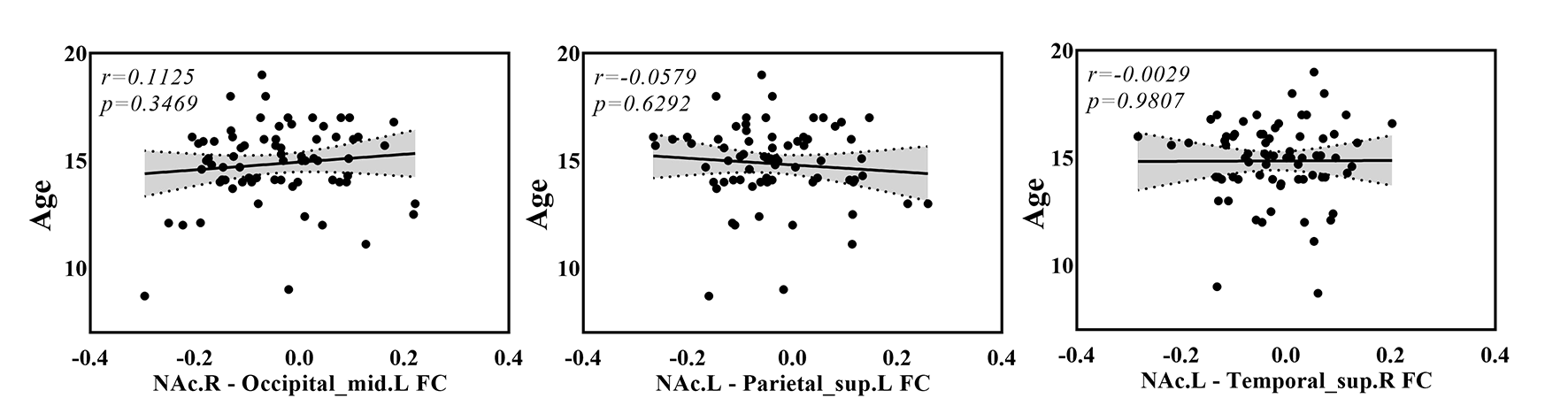

Supplement: Supplementary file 2 [file Image_2.TIF]
